# Prevention and Management of Diabetes-Related Foot Ulcers through Informal Caregiver Involvement: A Systematic Review

**DOI:** 10.1155/2022/9007813

**Published:** 2022-04-13

**Authors:** Joseph Ngmenesegre Suglo, Kirsty Winkley, Jackie Sturt

**Affiliations:** ^1^Florence Nightingale Faculty of Nursing, Midwifery and Palliative Care, Kings College London, UK; ^2^Department of Nursing, Presbyterian University College Ghana, Ghana

## Abstract

**Background:**

The literature remains unclear whether involving informal caregivers in diabetes self-care could lead to improved diabetic foot outcomes for persons at risk and/or with foot ulcer. In this review, we synthesized evidence of the impact of interventions involving informal caregivers in the prevention and/or management of diabetes-related foot ulcers.

**Methods:**

A systematic review based on PRISMA, and Synthesis Without Meta-analysis (SWiM) guidelines was conducted. MEDLINE (Ovid), Embase (Ovid), PsycINFO, CINAHL, and Cochrane Central Register of Controlled Trial of the Cochrane Library databases were searched from inception to February 2021. The following MESH terms were used: diabetic foot, foot ulcer, foot disease, diabetes mellitus, caregiver, family caregiver ,and family. Experimental studies involving persons with diabetes, with or at risk of foot ulcers and their caregivers were included. Data were extracted from included studies and narrative synthesis of findings undertaken.

**Results:**

Following the search of databases, 9275 articles were screened and 10 met the inclusion criteria. Studies were RCTs (*n* = 5), non-RCTs (*n* = 1), and prepoststudies (*n* = 4). Informal caregivers through the intervention programmes were engaged in diverse roles that resulted in improved foot ulcer prevention and/or management outcomes such as improved foot care behaviors, increased diabetes knowledge, decreased HbA1c (mmol/mol or %), improved wound healing, and decreased limb amputations rates. Engaging both caregivers and the person with diabetes in education and hands-on skills training on wound care and foot checks were distinctive characteristics of interventions that consistently produced improved foot self-care behavior and clinically significant improvement in wound healing.

**Conclusion:**

Informal caregivers play diverse and significant roles that seem to strengthen interventions and resulted in improved diabetes-related foot ulcer prevention and/or management outcomes. However, there are multiple intervention types and delivery strategies, and these may need to be considered by researchers and practitioners when planning programs for diabetes-related foot ulcers.

## 1. Introduction

Diabetes mellitus is among the top four noncommunicable diseases (NCD) targeted for action under the Sustainable Development Goals by the United Nations in 2015. Thus, all member countries are required to reduce premature death due to NCD by a third before 2030 [1]. Over 460 million people had diabetes in 2019, and this number has been estimated to rise to 578 million and 700 million by 2030 and 2045, respectively [2]. This high prevalence of diabetes and its complications puts pressure on global health expenditure. For instance, in 2017, the global health expenditure was estimated at over 7 billion USD with around 4 million diabetes related deaths [3].

One of the commonest and most debilitating complications of diabetes is diabetic foot ulcer (DFU) [4]. People with diabetes have a lifetime risk of up to 25% of developing DFU, and this greatly increases their chances of lower limb amputations [5]. This has made DFU the leading cause of nontraumatic amputations [5], and morbidity and mortality related to DFU are almost 50% over a five-year period [6].

The burden and debilitating effects of DFU reflect the need for strategic interventions to prevent and/or manage DFUs. Patient education, specialist care, clear referral pathways, use of multidisciplinary/professional teams, and other stringent interventions have significantly reduced foot ulcers and lower limb amputation (LLA) in developed countries over the past two decades [7–9]. For instance, to manage and/or prevent foot complications, the National Institute for Health and Care Excellence (NICE) recommends the use of health professional led multidisciplinary foot care service teams. The guideline stipulates that those persons with diabetes should be assessed for their risk of foot problems when diabetes is diagnosed and at least annually thereafter. Appropriate management and/or prevention services are then put in place based on the risk stratification of the patient [10]. Similarly, the International Working Group on the Diabetic Foot (IWGDF) in its evidence-based guidelines suggested that all preulcerative signs on the foot of persons with diabetes must be treated. The IWGDF further recommended that recurrent foot ulcers should be prevented through the provision of integrated foot care. This integrated foot care includes professional foot care, adequate footwear, and structured education about foot care [11].

Apparently, prevention of DFU requires persons with diabetes to engage in appropriate self-care behaviors relating to wearing off-loading footwear, exercise, diet, blood glucose monitoring, medication, and foot care [10–12]. Nevertheless, self-care behavior in the management of chronic conditions like diabetes is a complex phenomenon and impacted by multiple factors including but not limited to issues pertaining to problem solving skills, self-efficacy, and environment [13–15]. Thus, the social environment consisting of family members, friends, and significant others of persons with diabetes stands as one of the factors that can significantly influence individual's ability to manage diabetes-related foot ulcers (DFUs) at home [16–18]. Consequently, it has been suggested that diabetes self-management interventions should demonstrate active patient engagement and involvement of the caregivers of people with diabetes [18–20]. Involving informal caregivers (ICG) in caring for DFU is particularly important to achieving treatment goals especially in settings where family ties are strong [21], and it is also a cost-effective strategy [20, 22]. ICG refers to persons providing unpaid services to the patient and may include parents, children, spouse, friends, other relatives, or nonkin. They are sometimes called ‘family caregivers,, or ‘caregivers' [23]. Though mostly not formally trained, they feel they have a moral and social obligation to care for the sick at home [20].

The presence of ICG at home can play the role of negotiating and monitoring how well patients are following self-management plans [20], detecting any improvement or deterioration in patients' health situation while providing care and calling for medical assistance when needed [24]. The social support offered by ICGs also creates a feeling of acceptance and high level life satisfaction among persons with diabetes-related foot problems [25]. However, it has been identified that majority of ICG fear making mistakes and found tasks such as wound dressing to be emotionally challenging and indicated that they needed training to be effective at home [26]. Despite some of the known evidence of the active role ICGs can play in DFU care, a systematic review indicated that from 1995 to 2013, only 1% of publications in the literature mentioned ICGs as members of the wound care team [27]. It was identified in another study that 11% of ICGs are actively involved in the management of DFU and that interventions should be planned to include patients and their ICGs [19]. To effectively engage ICGs in DFU interventions, there is the need to synthesize the evidence to ascertain the impact of ICGs on the prevention and/or management of DFUs. Therefore, this review is aimed at the following:

1. Determine how informal caregivers' engagement in interventions can aid in the prevention and management of diabetes-related foot ulcers in adults
2. Understand the types of interventions participated in by informal caregivers to prevent and/or manage diabetes-related foot ulcers

## 2. Materials and Methods

This review was guided by the Preferred Reporting Items for Systematic Reviews and Meta-Analyses (PRISMA) [28] together with recommendations from the Synthesis Without Meta-analysis (SWiM) in systematic review guidelines [29]. The protocol was duly registered with the International Prospective Register of Systematic Reviews, PROSPERO, with registration number: CRD42021231768.

### 2.1. Eligibility Criteria

The review was based on predefined criteria for inclusion and exclusion of studies as indicated in Table 1.

### 2.2. Searching and Selection of Studies

The search for studies was conducted in five databases without recourse to publication date, country, or language. Using both subject headings and key words, a search strategy (see supplementary file 1) was constructed and optimized for each of the following databases from inception to February 2021: MEDLINE (Ovid), Embase (Ovid), PsycINFO, Cumulative Index of Nursing and Allied Health Literature (CINAHL), and Cochrane Central Register of Controlled Trial of the Cochrane Library. Additionally, the reference list of included studies and relevant systematic reviews were screened for studies that might have been missed by the searched strategy. All searched outcomes were imported into Covidence systematic review manager, and duplicates were automatically detected and removed. Titles and abstracts of the studies were then screened and studies not relevant to the aim of this review excluded. The full text of potentially eligible studies was read in full by two authors, and disagreements were discussed to reach consensus.

### 2.3. Quality Assessment and Data Extraction

The Cochrane Collaboration risk of bias for RCTs [30] and risk of bias in nonrandomized studies of interventions (ROBINS-I) [31] tools were used in assessing the studies. Certainty of the evidence was ranked using the Grading of Recommendations, Assessment, Development and Evaluations (GRADE) guidelines for no single or no pooled estimate of effect [32]. Rating was done using the GRADEpro GDT software (https://gdt.gradepro.org/app/#organizations).

Data extraction used a modified template in covidence which was first piloted with one study. Also, the Template for Intervention Description and Replication (TIDieR) checklist [33] was used to guide the extraction of the necessary components of various interventions in studies. Key data extracted from studies included study reference, objective, setting, sample for intervention and control groups, participants characteristics, postintervention follow-up time, intervention description, and relevant outcomes.

### 2.4. Data Synthesis

A narrative synthesis of findings guided by the SWiM guidelines [29] was undertaken due to high heterogeneity in included studies in their intervention types, duration, data collection time points, and settings which made meta-analysis inappropriate. Therefore, data was synthesized based on direction of effect. Based on the objectives of this review, the first stage of synthesis was done to determine how ICG interventions aided outcomes pertaining to the prevention and management of DFU, and the second stage evaluated the various types of ICG interventions utilized to prevent or manage DFU. Studies were grouped based on the outcomes reported, and these outcomes were subsequently grouped into DFU prevention outcomes and DFU management outcomes. The prevention outcomes included HbA1c, diabetes knowledge, and foot self-care behavior/practices, while the DFU management outcomes measured among persons with current DFU included wound healing and limb amputation. To facilitate description of the ICG interventions, study intervention types were coded as educational, behavioral, psychological, and mixed (psychobehavioral/educational) [34, 35]. Educational interventions were programs implemented by the health professional that focused on providing participants with information to enhance their knowledge of foot and diabetes self-management. Behavioral interventions focus on skills training, change in skills and lifestyle, aimed at improving self-management behaviors. Interventions were classed as psychological if their major aim were to address negative mood states, social support, and coping skills. Finally, interventions were described as mixed if they used two or more of the above categories of interventions.

## 3. Results of Review

### 3.1. Search Results

**Figure 1:**
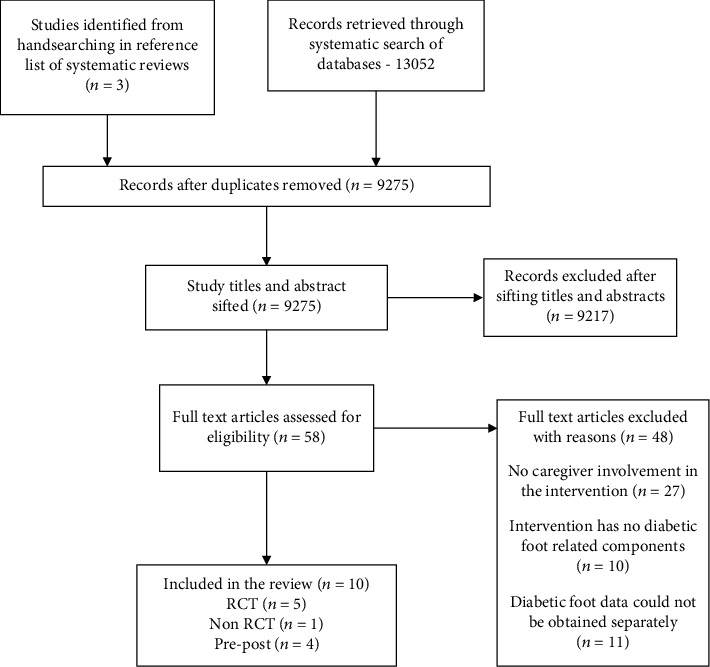
PRISMA flow diagram for study identification and selection process.

The primary search of databases identified seven eligible papers and several relevant systematic reviews. The reference list of systematic reviews was checked, and further three eligible papers were identified from three systematic reviews [36–38]. This resulted in ten primary studies being included in this review. The searching and selection of studies and reasons for excluding studies after full-text retrieval are presented in Figure 1, PRISMA flow diagram.

### 3.2. Characteristics of Included Studies

The review included 10 studies. Three of the studies came from the USA [39–41], two from China [42, 43], two from Indonesia [44, 45] and one each from Iran [46], Ireland [47], and India [48]. The total number of participants with diabetes was 5532. Only five studies [39, 41, 43–45] reported the characteristics and number of caregivers involved which was 359. Majority of caregivers were female (73.6%), and family relationship was mostly spouse or partner (53.6%), son/daughter (28.3%), or other family members (14.7%). Parents and siblings were the least likely to be involved as caregivers, 1.5% and 1.9%, respectively.  Table 2 presents the characteristics of studies.

### 3.3. Risk of Bias and GRADE Assessment

The risk of bias assessment of studies indicating authors judgement with supporting reasons are presented as supplementary files 2 and 3 for RCTs and non-randomized studies, respectively. All the RCTs had high risk of bias for nonblinding of participants and personnel since it was not possible to blind these people. Apart from one study [47], it was unclear if outcome assessors were blinded or not. Non-RCT studies were all rated moderate for bias due to confounding, and they all also had an overall moderate risk of bias. GRADE assessment for each outcome followed the criteria for evidence ranking in the absence of single estimate of effect [32]. Most outcomes were graded as moderate (see Table 3) due to serious risk of bias, serious inconsistency, but not serious indirectness and imprecision.

### 3.4. Outcomes and Measures

The first objective of this review was to determine how ICG interventions aided the prevention and management of DFUs. The outcomes reported by studies consisted of DFU prevention and DFU management outcomes as presented in Tables 3 and 4, respectively. Prevention outcomes reported by almost all studies included foot self-care behaviors/practices of participants, diabetes knowledge, and HbA1c [39–44, 46–48]. Wound healing and limb amputations/surgical interventions were the DFU management outcomes indicated by four studies [42, 44, 45, 48].

Seven studies assessed the foot self-care behavior and practices of participants, and six of them indicated improvement in foot care behavior of participants at follow-up [39–43, 46]. Five studies further indicated that the change in foot care practice was significant in the ICG intervention groups [40–43, 46]. The foot care activities engaged in by ICGs included assisting persons with diabetes in nail trimming, daily foot inspection, footwear inspection, checking of water temperature before patients washed their feet, checking of protective foot sensitivity using monofilaments, and collaborative problem solving. The assessment of participants' foot care behavior differed across studies. In majority of the outcome measure instruments, foot care questions were few, and only a part of a generic tool used in assessing participants' diabetes self-management activities [39–41, 46, 47]. However, two studies used diabetes foot self-care behavior scale (DFSCBS) and diabetes foot care scale (DFCS) that were specifically designed for assessing foot care practices [42, 43]. Both the generic diabetes self-management tools (summary of diabetes self-care activities scale (SDSCA)) and specifically devised foot care measure (DFCS) both recorded improved foot care practices among participants.

Study participants' knowledge on diabetes was assessed by three studies, and all of them reported significant improvement at postintervention follow-ups [40]–[42]. A supportive family member or friend was included in these interventions to encourage shared learning and to enhance the abilities of the ICG to know how to be helpful to the person with diabetes. Diabetes knowledge was assessed using either the Spoken Knowledge in Low Literacy patients with Diabetes (SKILLD) [40, 41] or Diabetes Knowledge Questionnaire (DKQ) [42]. Both outcome measure instruments suggested that interventions were effective in improving diabetes knowledge among participants [40–42].

Finally, under DFU prevention outcome, all ten included studies except one [43] investigated participants HbA1c at various time points' postintervention. Even though all nine studies reported reduction in HbA1c, almost equal number of studies, four and five reported insignificant and significant improvement, respectively, at postintervention follow-up. Measuring HbA1c at 3, 6, 12, or 18 months postintervention could still result in either significant or insignificant improvement in HbA1c results. The ICGs and persons with diabetes in the intervention groups were offered education on physical activity (exercise), blood glucose monitoring, healthy eating habits, and medication regimens. ICGs acted as support persons and helped in dietary planning and setting of diabetes management goals.

DFU management outcomes were assessed among participants who already had DFU problems. Healing of diabetic wounds was objectively assessed in three studies [44, 45, 48], and all of them reported clinically significant improvement in wound size. Limb amputations/surgical interventions prevalence was also recorded by two studies [42, 48]. These studies observed that even though the difference was not significant, amputations were lower in the study intervention group compared to study control group [42]. To actively support the management of DFU, ICGs in the intervention programs together with the person with DFU were trained on wound care. Family caregivers were taught their roles and effective involvement in DFU care, problem solving skills, and diet planning [42, 44, 45, 48].

### 3.5. Intervention Types

Various intervention types as operationally defined earlier were implemented to prevent and/or manage DFU. They included six psychobehavioral/educational type intervention [39–41, 44–46], two behavioral [42, 48] and one each of psychological [47] and educational interventions [43]. These interventions were delivered over several sessions with a mean of 15 (range 3 to 24) over a mean duration of 20 weeks (range 3 to 104) (see supplementary file 4 for coded intervention types and delivery methods).

The study intervention types implemented produced similar results on the outcomes measured. Apart from psychological intervention [47], all other intervention types reported improved diabetes knowledge and foot care behavior. These interventions were delivered through a mixture of didactic and interactive teaching methods, through face-to-face or phone calls. A mixed format of intervention delivery which involves a combined use of face-to-face, phone calls, videotapes and information booklets was utilized in behavioral or mixed psychobehavioral/educational interventions and resulted in significant improvement in foot self-care practices among participants [40–42, 45, 46]. ICGs and persons with diabetes were taught together in all interventions to promote shared learning and agreed self-care goals.

Behavioral interventions in China and India resulted in both improved foot care practices and lower prevalence of amputations [42, 48]. In these behavioral interventions, participants with diabetes and their caregivers were provided with skills training on various foot care activities and study participants tasked to report to clinic with any sign of foot disease for treatment. This intervention type even at long-term follow-up still recorded significant results at 12 months and 18 months, respectively, for Liang et al. [42] and Viswanathan et al. [48].

Also, behavioral [48] and mixed behavioral/educational interventions [44, 45] produced clinically significant reduction in diabetic wound size and healing time. Persons with diabetes and their ICGs in these interventions were engaged in participatory diabetes education, hands-on workshop on wound care, problem solving skills, and establishment of family roles in DFU care. Thus, an interactive and mixed method of teaching was utilized to achieve wound healing results. In these participatory teaching methods, diabetes self-management activities were discussed, and concerns of both the person with diabetes and their ICG were addressed before setting diabetes management goals [39–41, 46].

## 4. Discussion

### 4.1. Main Findings

This review is the first systematic review focusing solely on DFU and ICGs. It identified that trials of ICG interventions resulted in improved DFU prevention and management outcomes, possibly through the diverse roles played by ICGs. Thus, designing of interventions to engage family caregivers strengthened the programs, and this is evidenced in the improved foot self-care practices, improved diabetes knowledge, and better DFU management outcomes in the ICG intervention groups. Caregivers actively participated in the prevention of DFU through their diverse activities ranging from working collaboratively with the person with diabetes in feet inspection, checking of feet sensation, diet/meal planning, and setting of diabetes self-management goals. The management of DFUs was facilitated by ICGs through their engagement in wound care and participatory problem-solving activities. ICG participation in interventions characterized by hands-on skills training on wound and/or foot care, combined used of interactive, face-to-face and phone calls intervention delivery resulted in improved foot self-care behaviors and wound healing.

### 4.2. Findings Compared to Wider Evidence

The impacts of ICG interventions identified in this review are not dissimilar to other previous systematic reviews indicating that involvement of family caregivers in interventions improved clinical outcomes for persons with cancer, stroke, and other debilitating chronic conditions [21, 49–52]. For instance, an evidence synthesis involving stroke survivors indicated that family-oriented interventions were effective in reducing poststroke depression and improving the quality of life of both patients and caregivers [53]. Similar significant improved health outcomes were detected among persons with cancer and their family caregivers [51, 54]. Generally, the involvement of ICGs in the management of community-based adult is widely recommended as superior to patient-only interventions [55–57]. This probably is based on the assertion that family health and function influence the health status and functioning of individual family members, and a joint family and patient intervention could produce better health outcome for both. In the context of diabetes, our review findings resonates with previous systematic reviews suggesting that involvement of caregivers and social support significantly improves self-management behaviors and health outcomes of persons with diabetes [36, 58]. Our review reiterates the significance of ICG and the patient's social environment in the diabetes self-management continuum, and this could be applied in the prevention and management of diabetes-related foot ulcer. A review of reviews suggested that ICGs were often included in interventions and acted as a surrogate for the health care provider and the health care system. Family members were used as substitutes for professionals to deliver needed care, monitor, or encourage the patient to achieve desired health outcomes. These interventions were planned to strengthen family's ability to work together with the person with the chronic condition in solving challenging situations [21]. This consolidates our findings suggesting that ICGs were involved in setting diabetes management goals, diet planning, and other activities that strengthened the interventions and resulted in improved clinical outcomes. The skills and competence of these ICGs in our review were probably enhanced through the workshops and interactive sessions of the interventions. The need to train and engage ICGs in wound care process was further suggested in a national survey conducted in the United States. The survey reported that over a third of caregivers were providing wound care at home but indicated they were afraid of making mistakes and needed some skill training [59]. Therefore, the design of foot care programs could make family caregivers more confident in their support roles by incorporating easy-to-follow training for them. Nevertheless, even though none of the included studies critiqued or assessed how interventions affected ICG themselves, it is important that such programs prevent patient-caregiver conflicts by maintaining patient autonomy and reducing diabetes distress [20]. This might be necessary in maximizing the impact and sustainability of such ICG interventions.

This narrative synthesis further described the various types of interventions participated in by ICGs. Both persons with diabetes and their ICGs participated in interventions that were focused on providing problem-solving skills, foot care skills, and general diabetes information using diverse intervention delivery strategies. This seems to be consistent with previous study findings that education combined with specific behavioral change strategies produced improved health outcomes for persons with chronic conditions [35, 60, 61]. Our findings suggest that interventions that taught both patient and carer how to examine feet and provide foot-related care and wound care improved outcomes. Nevertheless, this does not corroborate with previous systematic reviews and meta-analysis suggesting that foot care education alone has no significant effect and that there is no advantage of combining different educational approaches in preventing/reducing DFU [62]. Another Cochrane review indicated that even though foot care knowledge and self-reported patient behavior seem to be positively influenced by education in the short term, there is insufficient robust evidence that patient education alone is effective in achieving clinically relevant reductions in ulcer and amputation incidence [9]. The differences in findings and the results of these systematic reviews [9, 62] must be viewed with caution as they reviewed educational intervention studies that focused primarily on the patient alone. However, this also suggest the need for future reviews to examine whether educational interventions engaging both patient and their ICG resulted in better DFU clinical outcomes compared with interventions targeting patients alone. This will reaffirm how and whether it is more beneficial to engage both persons with diabetes and their ICG when planning DFU programs.

### 4.3. Strengths and Limitations

This review is the first of its kind focusing solely on ICGs and DFU. It uses transparent and rigorous methods, following the PRISMA and SWiM guidelines, and this allows for reproducibility of this study. A limitation of this review is that, despite a comprehensive search strategy, eligible studies were identified from only six countries across the globe. This makes it unclear to what extend findings may be applicable to other dissimilar contexts. This indicates the dearth of literature in the field, and given the potential devastating impact of DFUs, more research is needed in other contexts and the findings integrated into appropriate health system response. Most outcomes on the GRADE evidence rating were ranked moderate due to risk of bias especially with baseline confounders in the quasiexperimental studies. Hence, subsequent studies should adapt a well-designed RCT approach to be able establish the exact impact of ICG interventions.

### 4.4. Recommendations for Practice, Research, and Policy

Based on the evidence of the roles ICGs play in diabetes-related foot ulcer prevention, health care practitioners ought to recognize carers as active members in DFU prevention and/or management strategies. This implies involving them in planning and determining diabetes management goals, establishing their specific roles and how they can be effectively involved in foot disease prevention and management. As part of diabetes self-management education and support (DSME/S), practitioners should take pragmatic efforts to enhance the knowledge, skills, and confidence of ICGs by organizing easy to do skills training and education for both carers and patients. Also, ICG involvement holds advantages in high and low resource settings and policymakers could optimize their health expenditure by supporting the involvement of this unpaid caring work by upskilling ICGs. There is evidence that involving both ICGs and patients in the management of chronic conditions is cost-effective and interventions produces long-lasting effects [20, 22]. Foot specialist services and other foot care resources are mostly either not available or not affordable to persons especially in developing countries. Involving ICGs could be an innovative health care intervention to prevent foot disease. It is therefore imperative that these interventions need evaluating in lower resource settings where the involvement of knowledgeable, skilled and confident ICGs could reap significant benefits to their family and community in the absence of access to high quality healthcare for people with and/or at risk of diabetic foot disease.

## Figures and Tables

**Table 1:** Eligibility criteria for studies.

PICOS	Inclusion	Exclusion
Participants	(i) Persons with diabetes type 1 or 2, with or without diabetes-related foot ulcer and their informal caregiver (ICG). (ii) Participants are persons 18 years and above. (iii) ICGs in this review were defined as parents, spouse, friends, significant other, or any unpaid person providing and/or assisting patient with care activities at home. (iv) The caregiver may or may not be residing with the patient but sees the patient on regular basis to assist with provision of care. (v) In studies where caregiver participation is only optional, then, the data for those who participated with their ICG (Dyad) must have been presented separately for such studies to be included.	(i) Persons with diabetes but resident in a nursing care home and hostels since the caregiver ratios and relationship will be dissimilar to a traditional home environment. (ii) Studies that not all participants participated with their caregivers and the data not presented separately for those who attended with their ICG. (iii) Studies involving only caregivers without patients and vice versa
Intervention	(i) Interventions or programs actively engaging patients and at least one component/session of the intervention involved the patient's ICG, aimed at preventing and/or managing DFUs. (ii) Prevention intervention refers to interventions or programs among persons with diabetes but without current DFU, and such interventions encourage or teach behaviors and practices that seeks to prevent patients from developing diabetes-related foot problems (see outcomes column). (iii) DFU management interventions pertain to interventions for persons with diabetes who have got current diabetes-related foot ulcer/problems and such interventions seeks to treat or avert the existing foot problems.	(i) Interventions involving only caregivers without patients (persons with diabetes) and vice versa.
Context	(i) Studies with their settings in hospitals, diabetic clinics, or communities in any part of the world.	(i) Nursing homes, care/residential homes and hostels where persons with diabetes are residing and cared for by carers and other employees.
Outcome	(i) Foot self-care behavior/practices (e.g., foot inspection, foot hygiene, nail care, appropriate footwear and socks, foot sensitivity checking, temperature checking., etc.). (ii) Knowledge on diabetes (iii) Glycated hemoglobin (HbA1c) (iv) Incidence of foot problems (e.g., incidence of primary and/or recurrent diabetic foot ulcers, diabetic neuropathies, limb amputations, foot disability, callus, and tinea pedis) (v) Wound size or wound healing	(i) Quality of life outcomes (ii) Cost-related outcomes
Type of studies	(i) Experimental design studies including randomized controlled trials (RCTs) and prepostdesign studies (ii) Cluster-RCTs and quasiexperimental studies (iii) Studies of any sample size, language, publication status, or setting were eligible	(i) Qualitative studies (ii) Reviews (iii) Methodological papers (iv) Noninterventional or observational studies

**Table 2:** Characteristics of studies.

Study ID & country	Study design; participants (I;C)	Participant characteristics	Intervention and control treatment		Intervention duration/follow up	Outcomes (I vs. C), (*P* value)
			I	C		
Liang et al. (2012), China [42]	RCT; I30; C29	**N** = 59 Mean age not stated Female *n* = 26	(i) Skills training and education on foot care through group diabetes classes, and provision of foot care kits. (ii) Delivered through hands-on workshops and telephone calls	Routine diabetes care and treatment which consisted of unstructured diabetes education	3 months 24 months	(i) HbA1c improved significantly (% mean ± SD) =6.5 ± 2.3 vs. 7.9 ± 4.2, (*P* = ˂0.01) (ii) More lower limb amputations were recorded in the control group; LLA (%) = 6.90 vs.0.00, (*P* = 0.4569) (iii) diabetes knowledge improved significantly (mean ± SD) =89.56 ± 7.00 vs 67.87 ± 5.26 (*P*˂0.01). (iv) Improved foot care behavior (mean ± SD) =87.24 ± 6.20 vs. 71.43 ± 5.17 (˂0.01)
Subrata et al. (2020), Indonesia [44]	RCT; I27; C29	*N* = 56 Mean age not stated Female n =20	(i) Self and family management support program. (ii) Delivered through skills training on wound care and motivational interviewing.	Routine diabetes care and unstructured education	3 months 3 months	(i) Improved HbA1c: MS = 10.92, *df* = 1, *F* = 6.65, *P* = 0.013 (values for I vs C not stated) (ii) Clinical and statistical improvement in wound size, mm^2:^MS = 21799.41, *df* = 1, *F* = 38.11, *P* = 0.000 (values for *I* vs. *R* not stated)
McEwen (2017), USA [39]	RCT; I83; C74	*N* = 157 Mean age 53.53 ± 9.0 Female *n* = 102	(i) Family-based self-management social support intervention, providing information on diabetes and complication prevention. (ii) Involved discussions to set diabetes management goals	Wait-list control	3 months 6 months	(i) Improved foot care behavior (mean ± SD) =5.91 ± 1.5 vs. 5.20 ± 2.0 (*P* = .50)
Maslakpak (2017), Iran [46]	RCT; I60; C30	*N* = 90 Mean age not stated Female *n* = 39	(i) Family-oriented empowerment diabetes education using face-to-face or telephone call. (ii) It involved collaborative problem solving and discussion with patients	Usual unstructured education and pamphlet	3 months 3 months	(i) Foot care behavior improved significantly for the intervention group (means ± SD) =28.99 ± 5.55 vs 11.23 ± 8.57, (*P* = 0.0001 (ii) Improved HbA1c (%) =1.2 ± 7.19 vs 1.5 ± 7.8 (*P* = 0.21)
Keogh et al. (2011), Ireland [47]	RCT; I60; C61	*N* = 121 Mean age not stated Female not stated	Individually tailored family education to address negative perceptions about diabetes using motivational interviewing techniques	Routine diabetes care without home visits	3 weeks 6 months	(i) Improved HbA1c (% mean ± SD) =8.41 ± 0.99 vs. 8.80 ± 1.36 (*P* = 0.04) (ii) Foot care behavior—reported there was no significant difference between groups, but no values provided
Appil et al. (2019), Indonesia [45]	Non-RCT; I17; C16	*N* = 33 Mean age not stated Female *n* = 19	(i) Family empowerment educational program to provide basic information on diabetes and foot ulcer care. (ii) Delivered though diabetes lectures and discussions	Nonstructural education from nurses	4 weeks 12 weeks	(i) Improved HbA1c (% mean ± SD) =8.81 ± 1.83 vs 10.40 ± 2.56 (*P* = .48) (ii) reported clinically significant improvement in wound size =4.71 ± 7.77 vs (*P* = .10)
Hu et al. (2014), USA [41]	Prepost; I36; C-	*N* = 36 Mean age 50 ± 11 Female *n* = 27	(ii) Family-based cultural intervention through group and family sessions to provide information on diabetes self-management (iii) It involved the use of picture illustrations, videotape stories and seminar discussions		10 weeks 4 weeks	(i) Improved HbA1c: Slope (95%CI) = −0.028 (−0.059 to 0.002), *P* = .0683 (ii) Improved foot self-care behavior: Slope (95% CI), *P* value = 0.242 (0.125 to 0.358), *P* = .0002 (iii) Significantly improved diabetes knowledge: slope (95% CI), P value =0.501 (0.389 to 0.614), *P*˂.0001
Williams et al. (2014), USA [40]	Prepost; I25; C-	*N* = 25 Mean age not sated Female *n* = 20	(i) Community group diabetes self-management education (DSME) based on the ADA self-care behavior (ii) This program used videotaped stories; assisting to set (i) This program used videotaped stories; assisting to set individual diabetes management goals and group discussions.		8 weeks 24 months	(i) Improved HbA1c (% mean ± SD) =7.40 (1.32), *P* = .26 (ii) Significantly improved foot care behavior (mean ± SD): 5.76 ± 1.76, (*P* = 0.001) (i) Significantly improved diabetes knowledge (mean ± SD): 76 ± 0.14, (*P* = 0.001)
Li et al. (2019), China [43]	Prepost; I80; C-	*N* = 80 Mean age = 64.91 ± 11.68 Female *n* = 42	(i) Foot self-care education using WeChat videos and telephone calls		Until discharge	(i) Significantly improved foot self-care behavior (mean ± SD) = 75.85 ± 5.04 (*P* = 0.000)
Viswanathan et al. (2005), India [48]	Prepost; I4872; C-	*N* = 4872 Mean age = 60.5 ± 8.8 Female *n* = 1450	(i) Intensive treatment of foot problems, support and education on foot care and foot checks. (ii) The intervention also involved the use of pictures of foot ulcers; provision of customized orthoses for patients and assisting patients to select appropriate footwears		Not stated 18 months	(i) HbA1c improved significantly at follow up (%mean ± SD) = 9.2 ± 2.1 vs 10.3 ± 3.3 (*P*˂0.0001) (ii) Wound healing improved significantly =48 ± 18 vs. 90 ± 27 (*P* = 0.0001) (iii) Surgical interventions reduced significantly *n*(%) = 23 (3) vs. 75 (14), (*P* = 0.0001)

I: intervention group; C: controlled group; SD: standard deviation; RCT: randomized controlled trial; non-RCT: nonrandomized controlled trial; LLA: lower limb amputation; PEDIS: Perfusion, Extent, Depth, Infection and Sensation; MS: mean square between subjects; %: percentage; HbA1c: glycated hemoglobin; vs.: versus; DSME: diabetes self-management education; ADA: American Diabetes Association.

**Table 3:** Diabetes-related foot ulcer prevention outcomes.

Outcomes	How outcome was measured	Results	Certainty of the evidence	Study IDs
(i) Foot care behavior/practices of participants	(i) DFCS (Liang et al.) (ii) SDSCA (Keogh et. al.; Maslakpak et al.; McEwen et al. (iii) SKILLD (William et al.) (iv) Revised SDSCA (Hu et al.) (v) DFSCBS (Li et al.)	Seven studies reported on the foot care behavior of participants. Six out of the seven studies recorded an improvement in foot self-care practices of participants at postintervention, and the difference with baseline scores in each study was significant.	⨁⨁⨁◯ Moderate	(i) Liang (2012) (ii) Keogh (2012) (iii) Maslakpak (2017) (iv) McEwen (2017) (v) Hu (2014) (vi) William (2014) (vii) Li (2019)
(i) Diabetes knowledge	(i) DKQ (Liang et al.) (ii) SKILLD (Hu et al.; Williams et al.)	Two studies had data on this outcome, and each of them indicated that knowledge on diabetes increased significantly in the intervention groups and was sustained at 1 year and 2 years follow-ups	⨁⨁⨁⨁ High	(i) Liang (2012) (ii) Hu (2014) (iii) William (2014)
(i) HbA1c	(i) DAC machine (McEwen et al.) (ii) Bayer A1C NOW kit (Hu et al.) (iii) Laboratory values used by all other studies	Nine studies contributed data to this outcome. All nine studies reporting the levels of HbA1c indicated that there was improvement in the level of HbA1c at postintervention. However, two out of the nine studies indicated that though there was improvement in the intervention group, the difference was not significant when compared with the baseline values	⨁⨁⨁◯ MODERATE	(i) Liang et al. (2012) (ii) Subrata et al. (2020) (iii) McEwen et al. (2017) (iv) Maslakpak et al. (2017) (v) Keogh et al. (2011) (vi) Hu et al. (2014) (vii) William et al. (2014) (viii) Viswanathan et al. (2005) (ix) Appil et al. (2020)

Key: DFSCBS: Diabetes Foot Self-care Behaviour Scale; SDSCA: Summary of Diabetes Self-care Activities scale; DFUAS: Diabetes Foot Ulcer Assessment Scale; PEDIS: Perfusion, Extent, Depth, Infection and Sensation; SKILLD: Spoken Knowledge in Low Literacy patients with Diabetes; DKQ: Diabetes Knowledge Questionnaire; DFCS: Diabetes Foot Care Scale.

**Table 4:** Diabetes-related foot ulcer management outcomes.

Outcomes	How outcomes were measured	Results	Certainty of evidence	Study IDs
Wound healing	(i) PEDIS classification (Subrata et al.) (ii) DFUAS (Appil et al.) (iii) Not stated (Viswanathan et al.)	All three studies measuring this outcome each reported that there was improved reduction in wound sizes in the intervention groups and the difference was statistically and clinically significant.	⨁⨁◯◯ Low	Subrata et al. (2020) Appil et al. (2020) Viswanathan et al. (2005)
Amputations/surgical interventions	Objectively assessed or counted by the researcher	Each of the two studies that reported this outcome suggested there were lower numbers of amputations and surgical interventions in the intervention groups. However, the difference between groups was not significant in one of the studies (Liang et al.)	⨁⨁◯◯ Low	Liang et al. (2012) Viswanathan et al. (2005)

Key: DFSCBS: Diabetes Foot Sel-care Behaviour Scale; SDSCA: Summary of Diabetes Self-care Activities scale; DFUAS: Diabetes Foot Ulcer Assessment Scale; PEDIS: Perfusion, Extent, Depth, Infection and Sensation; SKILLD: Spoken Knowledge in Low Literacy patients with Diabetes; DKQ: Diabetes Knowledge Questionnaire; DFCS: Diabetes Foot Care Scale.

## Data Availability

All data generated or analyzed during this study are included in this article and its supplementary information files.

## Supplementary Materials

Supplementary 1 Supplementary file 1: search strategy.

Supplementary 2 Supplementary file 2: risk of bias for RCTs.

Supplementary 3 Supplementary file 3: Risk of bias for non-RCTs.

Supplementary 4 Supplementary file 4: description of interventions.

Supplementary 5 Supplementary file 5: coded intervention elements.

Supplementary 6 PRISMA checklist.

## References

[1] Desa U. N. (2015). Transforming our world: the 2030 agenda for sustainable development. *Sustainable Development Knowledge Platform*.

[2] Saeedi P., Petersohn I., Salpea P. (2019). Global and regional diabetes prevalence estimates for 2019 and projections for 2030 and 2045: results from the International Diabetes Federation Diabetes Atlas, 9^th^ edition. *Diabetes Research and Clinical Practice*.

[3] International Diabetes Federation (2017). *IDF diabetes atlas*.

[4] McInnes A. D. (2012). Diabetic foot disease in the United Kingdom: about time to put feet first. *Journal of Foot and Ankle Research*.

[5] Boulton A. J., Vileikyte L., Ragnarson-Tennvall G., Apelqvist J. (2005). The global burden of diabetic foot disease. *Lancet*.

[6] Walsh J. W., Hoffstad O. J., Sullivan M. O., Margolis D. J. (2016). Association of diabetic foot ulcer and death in a population-based cohort from the United Kingdom. *Diabetic Medicine*.

[7] Komelyagina E. Y., Volkovoy A. K., Sanbanchieva N. I., Zaichikova M. F., Maksimov N. V., Antsiferov M. B. (2016). Multidisciplinary team approach for diabetic foot patients in an out-patient clinic. *Klinicheskaia Meditsina*.

[8] Monami M., Zannoni S., Gaias M., Nreu B., Marchionni N., Mannucci E. (2015). Effects of a short educational program for the prevention of foot ulcers in high-risk patients: a randomized controlled trial. *International Journal of Endocrinology*.

[9] Dorresteijn J. A., Kriegsman D. M., Assendelft W. J., Valk G. D. (2012). Patient education for preventing diabetic foot ulceration. *Cochrane Database of Systematic Reviews*.

[10] National Health and Medical Research Council (2019). *Diabetic foot problems: prevention and management*.

[11] Bus S. A., Lavery L. A., Monteiro-Soares M. (2020). Guidelines on the prevention of foot ulcers in persons with diabetes (IWGDF 2019 update). *Diabetes/Metabolism Research and Reviews*.

[12] Delamater A. M. (2006). Improving patient adherence. *Clinical Diabetes*.

[13] Ciechanowski P. S., Katon W. J., Russo J. E., Walker E. A. (2001). The patient-provider relationship: attachment theory and adherence to treatment in diabetes. *The American Journal of Psychiatry*.

[14] King D. K., Glasgow R. E., Toobert D. J. (2010). Self-efficacy, problem solving, and social-environmental support are associated with diabetes self-management behaviors. *Diabetes Care*.

[15] O’Dell K., O’Dell M. (2006). Socio-ecological resources for diabetes self-management. *Journal of the Mississippi State Medical Association*.

[16] Suglo J. N., Evans C. (2020). Factors influencing self-management in relation to type 2 diabetes in Africa: a qualitative systematic review. *PLoS One*.

[17] Diana G., Richard G., Guthrie D. W., Guthrie R. A. (2002). Nursing Management of Diabetes Mellitus. *A Guide to the Pattern Approach*.

[18] Isworo A., Ekowati W., Iskandar A., Latifah L. (2018). Family involvement programmes on the metabolic response of diabetic patients. *Health Science Journal*.

[19] Sinclair A. J., Armes D. G., Randhawa G., Bayer A. J. (2010). Caring for older adults with diabetes mellitus: characteristics of carers and their prime roles and responsibilities. *Diabetic Medicine*.

[20] Messenger G., Taha N., Sabau S., AlHubail A., Aldibbiat A. M. (2019). Is there a role for informal caregivers in the management of diabetic foot ulcers? A narrative review. *Diabetes Therapy*.

[21] Gilliss C. L., Pan W., Lindsey Davis L. (2019). Family involvement in adult chronic disease care: reviewing the systematic reviews. *Journal of Family Nursing*.

[22] Lee A. A., Piette J. D., Heisler M., Janevic M. R., Langa K. M., Rosland A. M. (2017). Family members’ experiences supporting adults with chronic illness: a national survey. *Families, Systems, & Health*.

[23] Triantafillou J. (2010). *Informal care in the long-term care system*.

[24] Reinhard S. C., Carol L., Sarah S. (2012). *Home Alone: Family Caregivers Providing Complex Chronic Care*.

[25] Brier M. J., Williams R. M., Turner A. P. (2018). Quality of relationships with caregivers, depression, and life satisfaction after dysvascular lower extremity amputation. *Archives of Physical Medicine and Rehabilitation*.

[26] Kirkland-Kyhn H., Generao S. A., Teleten O., Young H. M. (2018). Teaching wound care to family caregivers. *The American Journal of Nursing*.

[27] Moore Z., Butcher G., Corbett L. Q., McGuiness W., Snyder R. J., Van Acker K. (2014). Exploring the concept of a team approach to wound care: managing wounds as a team. *Journal of Wound Care*.

[28] Moher D., Liberati A., Tetzlaff J., Altman D. G. (2009). Preferred reporting items for systematic reviews and meta-analyses: the PRISMA statement. *BMJ*.

[29] Campbell M., McKenzie J., Sowden A. (2020). Synthesis without meta-analysis (SWiM) in systematic reviews: reporting guideline. *BMJ*.

[30] Higgins J. P., Altman D. G., Gøtzsche P. C. (2011). The Cochrane Collaboration’s tool for assessing risk of bias in randomised trials. *BMJ*.

[31] Sterne J. A., Hernán M. A., Reeves B. C. (2016). ROBINS-I: a tool for assessing risk of bias in non-randomised studies of interventions. *BMJ*.

[32] Murad M. H., Mustafa R. A., Schünemann H. J., Sultan S., Santesso N. (2017). Rating the certainty in evidence in the absence of a single estimate of effect. *BMJ Evidence-Based Medicine*.

[33] Hoffmann T. C., Glasziou P. P., Boutron I. (2016). Better reporting of interventions: template for intervention description and replication (TIDieR) checklist and guide. *Gesundheitswesen*.

[34] Sidani S., Braden C. (1998). *Evaluating nursing interventions: a theory-driven approach*.

[35] Fan L., Sidani S. (2009). Effectiveness of diabetes self-management education intervention elements: a meta-analysis. *Canadian Journal of Diabetes*.

[36] Baig A. A., Benitez A., Quinn M. T., Burnet D. L. (2015). Family interventions to improve diabetes outcomes for adults. *Annals of the New York Academy of Sciences*.

[37] van Netten J. J., Price P. E., Lavery L. A. (2016). Prevention of foot ulcers in the at-risk patient with diabetes: a systematic review. *Diabetes/Metabolism Research and Reviews*.

[38] Kodama S., Morikawa S., Horikawa C. (2019). Effect of family-oriented diabetes programs on glycemic control: a meta-analysis. *Family Practice*.

[39] McEwen M. M., Pasvogel A., Murdaugh C., Hepworth J. (2017). Effects of a family-based diabetes intervention on behavioral and biological outcomes for Mexican American adults. *The Diabetes Educator*.

[40] Williams I. C., Utz S. W., Hinton I., Yan G., Jones R., Reid K. (2014). Enhancing diabetes self-care among rural African Americans with diabetes. *The Diabetes Educator*.

[41] Hu J., Wallace D. C., Mccoy T. P., Amirehsani K. A. (2014). A family-based diabetes intervention for Hispanic adults and their family members. *The Diabetes Educator*.

[42] Liang R., Dai X., Zuojie L., Zhou A., Meijuan C. (2012). Two-year foot care program for minority patients with type 2 diabetes mellitus of Zhuang Tribe in Guangxi, China. *Canadian Journal of Diabetes*.

[43] Li J., Gu L., Guo Y. (2019). An educational intervention on foot self-care behaviour among diabetic retinopathy patients with visual disability and their primary caregivers. *Journal of Clinical Nursing*.

[44] Subrata S. A., Phuphaibul R., Grey M., Siripitayakunkit A., Piaseu N. (2020). Improving clinical outcomes of diabetic foot ulcers by the 3-month self- and family management support programs in Indonesia: a randomized controlled trial study. *Diabetes and Metabolic Syndrome: Clinical Research & Reviews*.

[45] Appil R., Sjattar E. L., Yusuf S., Kadir K. (2020). Effect of family empowerment on HbA1c levels and healing of diabetic foot ulcers. *The International Journal of Lower Extremity Wounds*.

[46] Hemmati Maslakpak M., Razmara S., Niazkhani Z. (2017). Effects of face-to-face and telephone-based family-oriented education on self- care behavior and patient outcomes in type 2 diabetes: a randomized controlled trial. *Journal of Diabetes Research*.

[47] Keogh K. M., Smith S. M., White P. (2011). Psychological family intervention for poorly controlled type 2 diabetes. *The American Journal of Managed Care*.

[48] Viswanathan V., Madhavan S., Rajasekar S., Chamukuttan S., Ambady R. (2005). Amputation prevention initiative in South India. *Diabetes Care*.

[49] Martire L. M. (2005). The "relative" efficacy of involving family in psychosocial interventions for chronic illness: are there added benefits to patients and family members?. *Families, Systems, & Health*.

[50] Torenholt R., Schwennesen N., Willaing I. (2013). Lost in translation--the role of family in interventions among adults with diabetes: a systematic review. *Diabetic Medicine*.

[51] Hopkinson J. B., Brown J. C., Okamoto I., Addington-Hall J. M. (2012). The effectiveness of patient-family carer (couple) intervention for the management of symptoms and other health-related problems in people affected by cancer: a systematic literature search and narrative review. *Journal of Pain and Symptom Management*.

[52] Hartmann M., Bäzner E., Wild B., Eisler I., Herzog W. (2010). Effects of interventions involving the family in the treatment of adult patients with chronic physical diseases: a meta-analysis. *Psychotherapy and Psychosomatics*.

[53] Vallury K. D. B., Jones M., Gray R. (2015). Do family-oriented interventions reduce poststroke depression? A systematic review and recommendations for practice. *Topics in Stroke Rehabilitation*.

[54] Griffin J. M., Meis L. A., MacDonald R. (2014). Effectiveness of family and caregiver interventions on patient outcomes in adults with cancer: a systematic review. *Journal of General Internal Medicine*.

[55] Chesla C. A. (2010). Do family interventions improve health?. *Journal of Family Nursing*.

[56] Rosland A. M., Piette J. D. (2010). Emerging models for mobilizing family support for chronic disease management: a structured review. *Chronic Illness*.

[57] Lyons K. S., Lee C. S. (2018). The theory of dyadic illness management. *Journal of Family Nursing*.

[58] Song Y., Nam S., Park S., Shin I.-S., Ku B. J. (2017). The impact of social support on self-care of patients with diabetes: what is the effect of diabetes type? Systematic review and meta-analysis. *The Diabetes Educator*.

[59] Nabuurs-Franssen M. H., Huijberts M. S. P., Nieuwenhuijzen Kruseman A. C., Willems J., Schaper N. C. (2005). Health-related quality of life of diabetic foot ulcer patients and their caregivers. *Diabetologia*.

[60] Roter D. L., Hall J. A., Merisca R., Nordstrom B., Cretin D., Svarstad B. (1998). Effectiveness of interventions to improve patient compliance. *Medical Care*.

[61] Litzelman D. K., Slemenda C. W., Langefeld C. D. (1993). Reduction of lower extremity clinical abnormalities in patients with non-insulin-dependent diabetes mellitus. *Annals of Internal Medicine*.

[62] Adiewere P., Gillis R. B., Imran Jiwani S., Meal A., Shaw I., Adams G. G. (2018). A systematic review and meta-analysis of patient education in preventing and reducing the incidence or recurrence of adult diabetes foot ulcers (DFU). *Heliyon*.

