# Supplementary material for: Prevention and Management of Diabetes-Related Foot Ulcers through Informal Caregiver Involvement: A Systematic Review
Source: J Diabetes Res. 2022 Apr 13;2022:9007813. doi: 10.1155/2022/9007813 (PMC9021995; doi:10.1155/2022/9007813)
Supplement: Supplementary 1 — Supplementary file 1: search strategy. [file 9007813.f1.docx]

**MEDLINE Search Strategy**

1. exp Diabetes Mellitus/
2. exp Foot Ulcer/
3. exp Diabetic Foot/
4. (diabet* adj3 ulcer*).tw.
5. (diabet* adj3 foot).tw.
6. (diabet* adj3 wound*).tw.
7. exp diabetic neuropathies/
8. (diabet* adj3 neuropath*)
9. exp Foot Diseases/
10. Or/2-9
11. 1 AND 10
12. exp Parents/
13. exp Family/
14. exp Caregivers/
15. ((parent$ or mother$ or father$) not parenteral$).tw.
16. (caregiver$ or care?giver$ or carer$ or guardian$).tw
17. (familial or families or friend$ or relative$ or spouse$ or partner$ or husband$ or wife or wives or child or children).tw.
18. exp Social Support/
19. exp Family Relations/
20. exp Family Nursing/
21. Or/12 -20
22. 11 AND 21
