# Supplementary material for: Prevention and Management of Diabetes-Related Foot Ulcers through Informal Caregiver Involvement: A Systematic Review
Source: J Diabetes Res. 2022 Apr 13;2022:9007813. doi: 10.1155/2022/9007813 (PMC9021995; doi:10.1155/2022/9007813)
Supplement: Supplementary 2 — Supplementary file 2: risk of bias for RCTs. [file 9007813.f2.docx]

**Supplementary file 2: RISK OF Bias assessment for RCTs**

| **Subrata et al (2020)** | | | | | | |
| --- | --- | --- | --- | --- | --- | --- |
| **Criteria** | | **Judgement** | | **Comments to support judgement** | | |
| Sequence generation | | low | | Computer generated randomization schedule was used | | |
| Allocation concealment | | Low | | Group allocation was concealed to participants and researchers | | |
| Blinding of participants and personnel for all outcomes | | High | | Participants were not blinded | | |
| Blinding of outcome assessors | | Unclear | | The researchers did the data collection themselves, but it was not stated if they were blinded or not | | |
| Incomplete outcome data for all outcomes | | low | | Data for all outcomes were reported in tables | | |
| Selective outcome reporting | | low | | Outcomes set out on registered study protocol were all reported | | |
| Other sources of bias | | Unclear | | It was unclear to tell how the analysis use accounted for confounding factors | | |
| **Study: Maslakpak et al (2017**) | | | | | | |
| Sequence generation | | | low | | Random allocation software was used to randomised participants | |
| Allocation concealment | | | low | | Randomisation was done with a software and the results were concealed from research team and participants | |
| Blinding of participants and personnel for all outcomes | | | High | | Participants could not be blinded to the intervention | |
| Blinding of outcome assessors | | | Unclear | | It was not stated who did the outcome data collection and whether the person was blinded | |
| Incomplete outcome data for all outcomes | | | low | | Outcome data for all outcomes were present | |
| Selective outcome reporting | | | low | | Outcomes as indicated in trial register were all reported | |
| Other sources of bias | | | Unclear | | It was not clear if the analysis adequately accounted for all confounding factors | |
| **McEwen et al (2017)** | | | | | | |
| Sequence generation | | | Unclear | | | Details of how the randomisation was done not provided in the published script. |
| Allocation concealment | | | Unclear | | | It was not clearly stated how this was ensured |
| Blinding of participants and personnel for all outcomes | | | High | | | Participants and those delivering the intervention were not blinded to the intervention. |
| Blinding of outcome assessors for all outcomes | | | Unclear | | | Not stated who collected the data and whether the person was blinded |
| Incomplete outcome data for all outcomes | | | low | | | Data on reported outcomes were complete |
| Selective outcome reporting | | | Unclear | | | Protocol for the study not published or registered to be able assess this |
| Other sources of bias | | | Unclear | | | Clear analysis plan and proptocol not available to able to determine if all outcomes were reported and confounding factors accounted for in analysis |
| **Liang et al 2012** | | | | | | |
| Sequence generation | | Unclear | | | Details of how the randomization was done not described | |
| Allocation concealment | | Unclear | | | Not stated if researchers and participants were concealed to group allocation | |
| Blinding of participants and personnel for all outcomes | | High | | | Participants could not be blinded | |
| Blinding of outcome assessors for all outcome | | Unclear | | | It was not stated who collected the outcome data whether the person was blinded | |
| Incomplete outcome data for all outcomes | | low | | | Outcome data was complete | |
| Selective outcome reporting | | unclear | | | Study protocol or registration was not available to assess this | |
| Other sources of bias | | unclear | | | Unable to tell because statistical analysis plan and study protocol not accessible | |
| **Keogh et al 20111** | | | | | | |
| Sequence generation | Low | | | | A remote computer-generated random number sequence was used to allocate participants | |
| Allocation concealment | low | | | | Allocation of participants to groups was done randomly after they had been recruited and completed their baseline data. Researcher and participants had to prior knowledge which group they will be allocated | |
| Blinding of participants and personnel for all outcomes | High | | | | Study subjects were not blinded to the intervention | |
| Blinding of outcome assessors for all outcomes | Low | | | | Outcome assessors had no knowledge of group allocation of participants | |
| Complete outcome data for all outcomes | Unclear | | | | Authors stated that outcomes were reported in tables, but the tables were not available e online | |
| Selective outcome reporting | Low | | | | All outcomes on study protocol were reported | |
| Other sources of biases | Unclear | | | | Tables cited in the published script were not accessible and they contain some outcome data that could not be assessed. | |
