# Supplementary material for: Prevention and Management of Diabetes-Related Foot Ulcers through Informal Caregiver Involvement: A Systematic Review
Source: J Diabetes Res. 2022 Apr 13;2022:9007813. doi: 10.1155/2022/9007813 (PMC9021995; doi:10.1155/2022/9007813)
Supplement: Supplementary 3 — Supplementary file 3: Risk of bias for non-RCTs. [file 9007813.f3.docx]

Supplementary file 3: Risk of Bias Assessment for nonrandomized studies

|  | Bias due to confounding | Bias in selection of participants into the study | Bias in classification of interventions | Bias due to deviations from intended interventions | Bias due to missing data | Bias in measurement of outcome | Bias in selection of the reported result | Overall |
| --- | --- | --- | --- | --- | --- | --- | --- | --- |
| Appil et al 2020 | moderate | low | Low | low | moderate | Moderate | No information | Moderate |
| William et al 2014 | moderate | Low | Low | low | moderate | Moderate | No information | moderate |
| Li et al 2019 | moderate | low | Low | low | moderate | Moderate | No information | moderate |
| Hu et al 2014 | moderate | low | Low | low | moderate | Moderate | No information | moderate |
| Viswanathan et al 2005 | serious | low | Moderate | low | moderate | Moderate | No information | Moderate |
