# Supplementary material for: Prevention and Management of Diabetes-Related Foot Ulcers through Informal Caregiver Involvement: A Systematic Review
Source: J Diabetes Res. 2022 Apr 13;2022:9007813. doi: 10.1155/2022/9007813 (PMC9021995; doi:10.1155/2022/9007813)
Supplement: Supplementary 4 — Supplementary file 4: description of interventions. [file 9007813.f4.docx]

**Description of interventions**

| **Study ID and country** | **Intervention type** | **Description of the intervention** | **Intervention provider and strategies** |
| --- | --- | --- | --- |
| Hu (2014)  USA | - Educational/behavioural | - A family-based cultural intervention. - 8-weekly session programme for persons with diabetes in groups and additional 2 family sessions for patients and their family members - Major topics covered included introduction to diabetes; exercise and foods; eating healthy; blood glucose monitoring; diabetes medication; Self-care; problem solving skills; coping strategies and action planning. | - A nurse practitioner/trained diabetes educator. - Used both individual (family sessions) and group session through face-to-face. - Family sessions were held at homes and group sessions with other patients at the study centre. - Programme delivered through use of picture illustrations; Videotape stories; Seminar discussions |
| Williams (2014)  USA | - psycho-behavioural/educational | - Community group DSME. - Group intervention sessions were loosely organised around topics of ADA Diabetes 7 self-care behaviours. - Interactive learning activities were based on the topic for each day after viewing a videotape story of an African American confronting a typical problem of self-management. | - Delivered by a CDE and nurse practitioner/case manager. - Through group discussions and problem solving of characters after videotape viewing. - Assisting participants to set individual diabetes management goals. |
| Keogh (2011)  Ireland | - Psychological | - Individually tailored family intervention - Intervention through motivational interviewing was individually tailored to address their specific problems and attempted to correct negative moods, perceptions about diabetes, examine how perceptions influence self-management, mobilize family support and develop personalised action plans | - Delivered by a health psychologist after receiving 16hours of training on motivational interviewing. - Delivered on a face-to-face at the participants home using motivational interviewing techniques |
| McEwen (2017)  USA | - psycho-educational | - Family-based self-management social support intervention. - Provision of information on managing diabetes to improve glycaemic control and prevent complications through food consumption, exercise and stress management - The 12 weeks of intervention consisting of six 2-hour educational and social support group session for 6weeks, three 2-hour home visits weekly for 3 weeks and three 20-minute telephone calls weekly for three weeks | - Delivered by a CDE (for the educational session) and a promotora (for the social support, home visits and phone calls) - Assisting participants to set diabetes self-management goals - Delivered on a face-to-face and on telephone - Done in groups and also individually for some sessions |
| Maslakpak (2017)  Iran | - psycho-behavioural intervention | - Family oriented empowerment diabetes education - Content of education covered appropriate diet, exercise, blood glucose monitoring, foot ulcer prevention and adherence to medication. - This also include listening to patient concerns and using collaborative problem-solving techniques. | - Intervention delivered by authors themselves. - Delivered face-to-face and on telephone - Some sessions were in groups and others done individually using collaborative problem solving |
| Li (2019)  China | - Educational | - Foot self-care education. - The intervention consisted of foot self-care education using education leaflets, DVD and WeChat videos | - Intervention delivered at the hospital by the primary nurses after receiving training. - It was done individually on a face-to-face combined with telephone reminders. - Nurses supervised the watching of WeChat Videos at the hospital |
| Viswanathan (2005)  India | - Behavioural | - Intensive treatment and patient education on diabetic foot. - Participants instructed and taken through regular foot examination, selection of footwear, individual counselling in the presence of family members on inspection of feet and visiting of clinic with foot problems. | - Interventionist not stated. - Delivered face-to-face - Use of pictures of foot ulcers to teach - Provision of customized orthoses for patients - Assisting patients to select appropriate footwears - Medical and surgical treatment of foot problems |
| Liang (2012)  China | - Behavioural | - Skills training and educational program on foot care. - After instruction on foot self-care, demonstration and return demonstration, each participant was tasked to perform daily foot care at home assisted by family members. Participants attended diabetes group classes every 3 to 6 months for knowledge reinforcement and foot care skills re-demonstration. | - Multidisciplinary team (endocrinologist, nurses and dietician) led by a diabetes specialist nurse. - Intervention delivered on face-to-face basis and telephone reminders - Provision of foot care tools kit to patients - Skills training on foot care |
| Appil (2019)  Indonesia | - Educational/behavioural | - Education on benefits of family empowerment, basic knowledge on diabetes, diet planning, medication, stress management, and caring for foot ulcers | - Interventionist not stated. - Delivered face-to-face, individually and in groups. - Individual sessions delivered at participants homes and group sessions done at the clinic. |
| Subrata (2020)  Indonesia | - Educational/behavioural | - Self- and family management support program. - Topics covered included physical activities, exercise, diet, medication, foot care and blood glucose control. The family management focussed on strengthening the family responsibility to deal with foot ulcer, establishing family roles in foot ulcer care, and active involvement in care | - Interventionist not stated - Delivered through skills training on wound care, Motivational interviewing and intensive health education - Delivered through face-to-face groups meetings |
