# Supplementary material for: Prevention and Management of Diabetes-Related Foot Ulcers through Informal Caregiver Involvement: A Systematic Review
Source: J Diabetes Res. 2022 Apr 13;2022:9007813. doi: 10.1155/2022/9007813 (PMC9021995; doi:10.1155/2022/9007813)
Supplement: Supplementary 5 — Supplementary file 5: coded intervention elements. [file 9007813.f5.docx]

**Coded Intervention Types and Delivery Strategies**

| Intervention elements | Number of studies |
| --- | --- |
| **Types of Interventions identified** | |
| Psychological | 1 |
| Educational | 1 |
| Behavioural | 2 |
| Mixed (combination of at least 2 of the above) | 6 |
| **Teaching methodology** | |
| Didactic only | 1 |
| Interactive only | 4 |
| Mixed (used both) | 5 |
| **Delivery Strategies** |  |
| Web-based/online only | 0 |
| Written documents only | 0 |
| Video | 1 |
| Face-to-face only | 2 |
| Phone calls only | 0 |
| Mixed, at least two of the above | 7 |
| **Format** | |
| One-to-one sessions only | 4 |
| Group sessions only | 3 |
| Mixed one-to-one and groups | 3 |
| **Number of intervention sessions delivered** | |
| ≤5 sessions | 2 |
| 6 – 10 | 2 |
| ≥11 | 6 |
| **Duration of intervention** | |
| ≤6weeks | 2 |
| 7-12 weeks | 6 |
| ≥13 weeks | 1 |
